# Supplementary material for: Uncovering the Mechanisms of Active Components from Toad Venom against Hepatocellular Carcinoma Using Untargeted Metabolomics
Source: Molecules. 2022 Nov 10;27(22):7758. doi: 10.3390/molecules27227758 (PMC9694973; doi:10.3390/molecules27227758)
Supplement: Supplementary file 1 [file molecules-27-07758-s001.zip › molecules-1981746-supplementary.pdf]

Uncovering the Mechanisms of Active Components from Toad Venom against Hepatocellular Carcinoma Using Untargeted Metabolomics

Pan Liang [1,2,3], Yining Ma [1,3], Luyin Yang [1,3], Linshen Mao [1,3], Qin Sun [1,3], Changzhen Sun [1,3], Zengjin Liu [1,3], Maryam Mazhar [1,3], Sijin Yang [1,2,3,*] and Wei Ren [1,3,*]

[1] National Traditional Chinese Medicine Clinical Research Base, Drug Research Center of Integrated Traditional Chinese and Western Medicine, The Affiliated Traditional Chinese Medicine Hospital of Southwest Medical University, Luzhou 646000, China
[2] State Key Laboratories for Quality Research in Chinese Medicines, Faculty of Chinese Medicine, Macau University of Science and Technology, Macau 853, China
[3] Institute of Integrated Chinese and Western Medicine, Southwest Medical University, Luzhou 646000, China

Experimental Section

In vivo compounds identification of toad venom in rat plasma

Toad venom extract with the concentration of 1 mg/mL was prepared according to our previous report [13]. Male Sprague-Dawley (SD) rats weighted 200-220 g were purchased from Beijing Vital River Laboratory Animal Technology Co., Ltd. (Beijing, China) and housed at constant temperature and humidity environment. Before the experiment, the rats were fasted for 12 h. After taking blank blood, rats were orally administrated toad venom extract with the dose of 300 mg/kg. The blood samples were collected from the eye sockets of rats after 0.5, 1, 2, 4, 6, 8, 12, and 24 h, respectively. Subsequently, all blood samples were immediately centrifuged at 4000 rpm for 10 min at 4 °C, and the upper plasma were collected and stored at -80 °C. Further, 100 μL medicated plasma obtained from each time point were mixed and added with 3-fold methanol to precipitate protein. Then, the mixture was centrifuged at 12000 rpm for 20 min at 4 °C. The supernatant was blow-dried with nitrogen and subsequently redissolved with 100 μL methanol/water solution (1:1, v/v). Meanwhile, blank plasma was also treated with same procedure.

UHPLC-MS/MS detection conditions of toad venom in rat plasma

The ultimate 3000 hyperbaric LC system connected with high-resolution Q-Exactive Orbitrap mass system (UHPLC-HR-Q-Exactive Orbitrap MS, Thermo Fisher Scientific, USA) was applied for qualitative analysis of compounds. In chromatographic system, the CSH C18 column (2.1*100 mm, 1.7 μm, Waters) was used for the separation of chemical compounds in samples. The column temperature was 35 °C. The mobile phase was composed of solvent A (water containing 0.1% formic acid) and B (acetonitrile). At a 0.1 mL/min flow rate, the injection volume was kept at 3 μL. The gradient elution conditions optimized: 0-3 min, 2% B; 3-5 min, 2-28% B; 5-12 min, 28% B; 12-22 min, 28-35% B; 22-30 min, 35-100% B; 30-32 min, 100% B; 32-35 min, 2%B.

The parameters of mass spectrometry in positive mode were optimized as follows. Capillary voltage and temperature: 3 kV, 320 °C; sheath gas flow: 30; aux gas flow: 5; Mass scan range: m/z 100-1500 Da, resolution: 30000. All MS data were imported into Xcalibur 4.4 software to complete the extraction of chromatographic peaks and fragmentation behavior information. Mass error was set as less than 5 ppm.

H&E staining

H&E staining were used to detect the cell morphology after treatments. The excised tumors were fixed in tissue fixative for at least 24 h and sliced after embedded with paraffin. Then the slices were observed under microscope (CIC, XSP-C204) and photographed randomly at 200-fold field.

Ki-67 staining

The excised tumors were fixed in tissue fixative over 24 h and sliced after embedded with paraffin. The cell proliferation of tumor slices was evaluated by Ki-67 staining. The primary antibody was Ki-67 monoclonal antibody (dilution 1:1000, Chengdu Lilai Biotechnology Co., Ltd.), and HRP-labeled goat antirabbit lgG (dilution 1:3000, Chengdu Lilai Biotechnology Co., Ltd.) was used as the secondary antibody. The blue nuclei of total normal cells were stained by hematoxylin and the brown nuclei of proliferated cells were stained by DAB chromogenic agent.

TUNEL staining

Tumor tissues were fixed, embedded, sliced, and performed by using TUNEL staining kit according to the product introduction manual.

Table S1. MS2 fragment of 27 compounds from toad venom extract detected in rat plasma.

No	Type	Chemical formula	Error (ppm)	[M+H]+ measured mass	MS^2 fragments	Compound
1	M	$C_{19}H_{27}O_7N_2$	-0.399	395.18112	371.0842、336.10825、**219.14949**、160.07590	Bufotenidine-5-O-glucuronide
2	P	$C_{13}H_{19}ON_2$	-0.020	219.14917	189.10678、**160.07587**、142.72940	Bufotenidine
3	P	$C_{13}H_{25}O_5N_4$	-1.060	317.18185	299.04590、289.06152、**271.05081**、264.13458、239.13916、175.11900	Pitneloyl arginine
4	P	$C_{14}H_{27}O_5N_4$	-0.533	331.19702	314.17133、296.16074、**278.15018**、250.15533、175.11903、158.09248	Suberoyl arginine
5	M	$C_{24}H_{33}O_7$	-1.546	433.22128	**415.21231**、397.20132、379.19077、361.18030、351.19583、333.18530	1-hydroxylarenobufagin
6	M	$C_{24}H_{33}O_7$	-1.477	433.22131	415.21191、397.20108、379.19067、**361.18027**、351.19565、333.18515	5-hydroxylarenobufagin
7	M	$C_{24}H_{33}O_7$	-1.200	433.22153	**415.21216**、397.20117、379.19064、351.19577、333.18524	16-hydroxylarenobufagin
8	M	$C_{24}H_{35}O_7$	-0.490	435.23709	417.22852、399.21710、**381.20654**、363.19620、345.18542、317.19049、285.18533	Hydratalbufarenogin
9	P	$C_{24}H_{35}O_6$	-1.491	419.24234	401.22281、383.22287、**365.21228**、347.20157、269.19019、251.17963	11α-hydroxytelocinobufagin
10	P	$C_{24}H_{33}O_6$	-1.522	417.22638	**399.21680**、381.20599、363.19565、353.21115、335.20068、317.19000、255.10147、213.16382	Ψ-Bufarenogin
11	P	$C_{24}H_{35}O_5$	-1.837	403.24756	385.23831、367.22766、**349.21701**、321.22202、253.19568	Gamabufotalin
12	P	$C_{24}H_{33}O_6$	0.539	417.22726	399.21732、381.20679、**363.19623**、345.18558、335.20120、249.16418	Bufarenogin
13	P	$C_{24}H_{35}O_5$	-0.101	403.24799	385.2384、367.22781、**349.21713、** 331.20660、253.19582	19-hydroxylbufalin
14	P	$C_{24}H_{33}O_6$	-1.522	417.22733	**399.21689**、381.20621、363.19574、335.20081、317.19022、289.19516、255.10167、213.16386	Arenobufagin
15	P	$C_{24}H_{35}O_5$	-0.324	403.24774	385.23831、367.22766、**349.21701**、271.20624、253.19569、215.17986	Desacetylbufotalin
16	P	$C_{24}H_{35}O_6$	0.035	419.24289	**401.23331**、383.22275、365.21213、347.20154、269.19077、213.16432	Hellebrigenol
17	P	$C_{24}H_{33}O_6$	-0.132	417.22711	399.21759、381.20703、363.19641、**335.20139**、213.16420	Hellebrigenin
18	P	$C_{24}H_{31}O_6$	-1.241	415.21106	397.20142、379.19083、**351.19595**、333.18546、283.16968、237.16400	Bufotalinin
19	P	$C_{24}H_{35}O_5$	-0.101	403.24774	385.23828、**367.22769**、349.21713、253.19576、215.1799	Telocinobufagin
20	P	$C_{26}H_{35}O_7$	-0.784	459.23801	**441.25177**、381.20599、363.19638、345.18564、335.20007	Cinobufaginol
21	P	$C_{24}H_{33}O_5$	-0.901	401.23135	383.22241、**365.21188**、347.20129、337.21701、251.17996、215.17982	Desacetylcinobufagin
22	P	$C_{24}H_{35}O_4$	-1.178	387.25244	369.24249、**351.23184**、333.22125、305.22629、255.21085	Bufalin
23	P	$C_{24}H_{33}O_4$	-0.950	385.23697	**367.22766**、349.21704、339.23270、321.22208、253.19574	Resibufogenin

24	P	$C_{26}H_{35}O_6$	-0.265	443.24225	401.23254、383.22198、**365.21167**、347.20099、319.20587、251.17960、215.17966	Cinobufagin
25	M	$C_{17}H_{27}O_2$	-1.013	263.20032	**245.19022**、227.17964、203.17963、161.13255、149.13257	Unknown
26	M	$C_{31}H_{41}O_{12}$	-0.076	605.45453	**587.44604**、569.43524、551.42450、493.33142、457.30991、251.17946	Cinobufagin-3-O-glucuronide
27	M	$C_{20}H_{33}O_2$	-1.824	305.24695	**287.23721**、269.22653、249.18501、235.16943、221.15375、177.16383、121.10121	Unknown

Table S2. Details of differential metabolites of HepG2 cell xenograft tumor among different groups compared with control group.

No	Groups	adduct	Name	VIP	Fold change	*p*-value	SuperClass	Class	Trend
1	Buf vs NS	[M+H]+	3-methylhistidine	3.776726251	2.497261661	0.000154606	Organic acids and derivatives	Carboxylic acids and derivatives	up
2	Buf vs NS	[M+H]+	Hyocholic acid	1.56194025	0.748862457	0.003292416	Lipids and lipid-like molecules	Steroids and steroid derivatives	down
3	Buf vs NS	[M+H-C6H11NO]+	L-leucyl-l-leucine methyl ester	1.104936126	1.398030724	0.010002956	Organic acids and derivatives	Carboxylic acids and derivatives	up
4	Buf vs NS	[M+H]+	Pipamperone	1.011033602	1.262223386	0.010254865	Organic oxygen compounds	Organooxygen compounds	up
5	Buf vs NS	(M+H)+	1-Methylhistamine	3.455060185	0.618015207	0.012391568	Organic nitrogen compounds	Organonitrogen compounds	down
6	Buf vs NS	[M+H]+	Niacinamide	28.9245572	1.199755098	0.014129786	Organoheterocyclic compounds	Pyridines and derivatives	up
7	Buf vs NS	[M+H]+	Mandelonitrile	5.083777088	1.54404592	0.015729502	Benzenoids	Benzene and substituted derivatives	up
8	Buf vs NS	[M+H]+	Heptadecasphinganine	3.998450845	1.281201527	0.02007736	Organic nitrogen compounds	Organonitrogen compounds	up
9	Buf vs NS	[M+H]+	5-methylcytidine	4.745699278	1.625335873	0.020545094	Nucleosides, nucleotides, and analogues	Pyrimidine nucleosides	up
10	Buf vs NS	(M+H-H2O)+	Succinic semialdehyde	1.670977384	1.184395409	0.027427747	/	/	up
11	Buf vs NS	[M+H]+	7-hydroxymitragynine	1.342687078	0.585678416	0.029686138	/	/	down
12	Buf vs NS	(M+H)+	S-Methyl-5’-thioadenosine	4.827334597	1.218389952	0.031278023	Nucleosides, nucleotides, and analogues	5’-deoxyribonucleosides	up
13	Buf vs NS	[M+H]+	Nudifloramide	5.671360515	1.6070879	0.034522412	Organoheterocyclic compounds	Pyridines and derivatives	up
14	Buf vs NS	[M+H-2H2O]+	Taurocholate	1.703227519	0.515168114	0.035610287	Lipids and lipid-like molecules	Steroids and steroid derivatives	down
15	Buf vs NS	[M+H-CH5N3]+	Arcaine	1.014612337	0.745443772	0.041093046	Organic nitrogen compounds	Organonitrogen compounds	down
16	Buf vs NS	[M+H]+	Pantetheine	1.147202604	1.677796315	0.042119083	Organic acids and derivatives	Carboxylic acids and derivatives	up
17	Buf vs NS	[M+H]+	Pro-Trp	4.744679355	0.458015185	0.044631654	Organic nitrogen compounds	Organonitrogen compounds	down
18	Buf vs NS	[M-H]-	Pg 34:2	1.282527768	1.333009097	0.02519202	Lipids and lipid-like molecules	Glycerophospholipids	up
19	Buf vs NS	[M-H]-	Dihydroxyacetone	1.756688698	1.885508052	0.033042166	Organic oxygen compounds	Organooxygen compounds	up
20	Buf vs NS	[M-H-NH3]-	1,2-dioleoyl-sn-glycero-3-phospho-l-serine	1.642912175	1.424214886	0.038186197	Lipids and lipid-like molecules	Glycerophospholipids	up
21	Cino vs NS	[M+Na]+	Carbobenzyloxy-l-norvalyl-l-norleucine	1.358516985	0.334816775	2.51373E-05	Organic acids and derivatives	Carboxylic acids and derivatives	down
22	Cino vs	[M+H-C7	Benzamide	2.4906731	0.71193622	0.0001597	Benzenoids	Benzene and	down

	NS	H4O2]+		99	5	71		substituted derivatives	
23	Cino vs NS	[M+H]+	7-hydroxymitragynine	1.726065093	0.388817251	0.001310844	/	/	down
24	Cino vs NS	[M+H-CH4O3]+	Trans-traumatic acid	1.228856273	0.802101086	0.0046496	Lipids and lipid-like molecules	Fatty Acyls	down
25	Cino vs NS	[M+H]+	Hyocholic acid	1.334863574	0.75217707	0.007237167	Lipids and lipid-like molecules	Stero/ids and steroid derivatives	down
26	Cino vs NS	[M+H]+	Pentaethylene glycol	1.681442078	1.525547777	0.008886735	Organic oxygen compounds	Organooxygen compounds	up
27	Cino vs NS	M+	Trigonelline	4.502426392	1.320183238	0.009900842	Alkaloids and derivatives		up
28	Cino vs NS	M+	1-Methylnicotinamide	12.07074518	0.819617274	0.010302756	Organoheterocyclic compounds	Pyridines and derivatives	down
29	Cino vs NS	[M+H]+	Zectran	2.558000856	1.226754954	0.019773551	Benzenoids	Benzene and substituted derivatives	up
30	Cino vs NS	[M+H]+	Tris(hydroxymethyl)aminomethane	1.597593336	1.129812923	0.023314582	Organic nitrogen compounds	Organonitrogen compounds	up
31	Cino vs NS	[M+H-C6H8O]+	1-(cyclopentylcarbonyl)-4-piperidinamine	1.332984509	0.327797998	0.026258287	Organoheterocyclic compounds	Piperidines	down
32	Cino vs NS	[M+H]+	Erucamide	4.268245997	1.349486659	0.040155258	Lipids and lipid-like molecules	Fatty Acyls	up
33	Cino vs NS	[M+H-C4H11N]+	Diethylcarbamazine	1.274715377	1.591059019	0.043129917	Organoheterocyclic compounds	Diazinanes	up
34	Mix vs NS	[M+Na]+	Carbobenzyloxy-l-norvalyl-l-norleucine	1.427974828	0.201760278	4.45537E-06	Organic acids and derivatives	Carboxylic acids and derivatives	down
35	Mix vs NS	(M+H)+	1,2-dioleoyl-sn-glycero-3-phosphatidylcholine	6.284527652	0.824330903	6.52656E-05	/	/	down
36	Mix vs NS	[M+H]+	7-hydroxymitragynine	1.893446977	0.168298037	0.000145617	/	/	down
37	Mix vs NS	[M+H-C7H13NO2]+	Dodecanoic acid	2.011373384	1.391568071	0.000148739	Lipids and lipid-like molecules	Fatty Acyls	up
38	Mix vs NS	[M+H-3H2O]+	3-hydroxydodecanoic acid	1.506076693	1.509538426	0.000238821	Organic acids and derivatives	Hydroxy acids and derivatives	up
39	Mix vs NS	[M+H-NH3]+	Phenylethylamine	4.697021056	1.373368003	0.000920221	Benzenoids	Benzene and substituted derivatives	up
40	Mix vs NS	[M+H-C11H17O2N]+	Benzeneethanamine	3.062304856	1.376871326	0.001147506	Benzenoids	Benzene and substituted derivatives	up
41	Mix vs NS	[M+H]+	4'-o-.beta.-d-glucosyl-5-o-methylvisamminol	1.446148541	1.629351405	0.001235892	Organoheterocyclic compounds	Benzopyrans	up
42	Mix vs NS	(M+H-H2O)+	3-Amino-3-(4-hydroxyphenyl)propanoate	1.051752504	1.851242739	0.001656808	/	/	up
43	Mix vs NS	[M+H]+	3,4-dimethylbenzaldehyde	2.213559503	1.365042922	0.002620174	Benzenoids	Benzene and substituted derivatives	up
44	Mix vs NS	[M+H-H2O]+	Benzyl alcohol	1.721799434	1.281453221	0.002644125	Benzenoids	Benzene and substituted derivatives	up
45	Mix vs NS	[M+H-C6H12O]+	Zerumbone	4.29158083	1.389182549	0.002723672	Lipids and lipid-like molecules	Prenol lipids	up
46	Mix vs NS	[M+H]+	Norvaline	1.856377978	1.285771303	0.002997729	Organic acids and derivatives	Carboxylic acids and derivatives	up
47	Mix vs NS	[M+H]+	2,5-di-tert-butyl-1,4-benzoquinone	1.790282994	2.068603292	0.003331879	Organic oxygen compounds	Organooxygen compounds	up

48	Mix vs NS	[M+H]+	Ropinirole	1.594695868	1.49244996	0.003657843	Organoheterocyclic compounds	Indoles and derivatives	up
49	Mix vs NS	[M+H]+	Eprosartan	1.48355726	1.537431138	0.004627101	Benzenoids	Benzene and substituted derivatives	up
50	Mix vs NS	[M+H-H2O]+	1-stearoyl-2-linoleoyl-sn-glycerol	1.312585783	0.761847751	0.006412221	Lipids and lipid-like molecules	Fatty Acyls	down
51	Mix vs NS	[M+H]+	Heptadecasphinganine	4.675512738	1.485856454	0.006496494	Organic nitrogen compounds	Organonitrogen compounds	up
52	Mix vs NS	[M+H]+	1-methyl-4-benzylpiperazine	1.16426828	1.542649532	0.009278051	Benzenoids	Benzene and substituted derivatives	up
53	Mix vs NS	[M+H-C6H11NO]+	L-leucyl-l-leucine methyl ester	1.832869864	1.877710068	0.010585838	Organic acids and derivatives	Carboxylic acids and derivatives	up
54	Mix vs NS	[M+H-H2O]+	Exo-norborneol	1.171385406	1.183553817	0.010613104	Lipids and lipid-like molecules	Prenol lipids	up
55	Mix vs NS	[M+H]+	L-serine methyl ester	1.747456322	1.722573203	0.01118915	Organic acids and derivatives	Carboxylic acids and derivatives	up
56	Mix vs NS	[M+H]+	Leu-Ala	1.727767618	2.565314868	0.011336122	Organic acids and derivatives	Carboxylic acids and derivatives	up
57	Mix vs NS	[M+H]+	Methyltyrosinate	1.612034157	2.505680244	0.012347015	Organic acids and derivatives	Carboxylic acids and derivatives	up
58	Mix vs NS	[M+H]+	α-linolenic acid	2.122515214	1.58454642	0.016772588	Lipids and lipid-like molecules	Fatty Acyls	up
59	Mix vs NS	[M+H]+	5-methylcytidine	6.013456579	2.19578021	0.020652219	Nucleosides, nucleotides, and analogues	Pyrimidine nucleosides	up
60	Mix vs NS	[M+NH4]+	9,12-octadecadiynoic acid	2.874831884	1.754476457	0.022890149	Lipids and lipid-like molecules	Fatty Acyls	up
61	Mix vs NS	[M+H]+	Benzimidazole	1.02167982	1.176313559	0.022891643		/	up
62	Mix vs NS	[M+H]+	Melamine	1.161084789	1.690624866	0.027740005	Organoheterocyclic compounds	Triazines	up
63	Mix vs NS	[M+H]+	Pipamperone	2.287747858	2.37423784	0.027790609	Organic oxygen compounds	Organooxygen compounds	up
64	Mix vs NS	[M+H]+	Chenodeoxycholate	1.480009647	2.14394956	0.037745367	Lipids and lipid-like molecules	Steroids and steroid derivatives	up
65	Mix vs NS	[M+H-2H2O]+	17-octadecynoic acid	1.086115355	1.220153034	0.041789498	Lipids and lipid-like molecules	Fatty Acyls	up
66	Mix vs NS	[M+H]+	Erucamide	3.670870785	1.534737462	0.047096049	Lipids and lipid-like molecules	Fatty Acyls	up
67	Mix vs NS	[M-H]-	Valeric acid	2.663097486	2.660455077	0.000214786	Lipids and lipid-like molecules	Fatty Acyls	up
68	Mix vs NS	[M-H]-	Thymol-beta-d-glucoside	5.384681197	1.878799394	0.001766187	Lipids and lipid-like molecules	Prenol lipids	up
69	Mix vs NS	[M-H]-	[(4e)-7-acetyloxy-6-hydroxy-2-methyl-10-oxo-2,3,6,7,8,9-hexahydrooxecin-3-yl] (e)-but-2-enoate	1.122046262	1.806634148	0.00192566	Organic acids and derivatives	Carboxylic acids and derivatives	up
70	Mix vs NS	[M-H]-	18-carboxydinorleukotriene b4	1.049090033	1.906441424	0.002839324	Lipids and lipid-like molecules	Fatty Acyls	up
71	Mix vs NS	[M-H]-	Ostruthin	2.190803297	1.9379619	0.003433409	Lipids and lipid-like molecules	Prenol lipids	up
72	Mix vs NS	[M-H]-	16-phenoxytetranorprostaglandin a2	1.717056289	2.233180547	0.003732374	Lipids and lipid-like molecules	Fatty Acyls	up
73	Mix vs NS	[M-H]-	Gly-His-Lys	9.151828421	1.786105705	0.005017745	Organic acids and derivatives	Carboxylic acids and derivatives	up
74	Mix vs NS	[M-H]-	Acetic acid	2.480761818	1.988144591	0.005079202	Benzenoids	Benzene and substituted	up

									derivatives	
75	Mix vs NS	[M-H]-	Propanoic acid	1.297956897	2.037459011	0.00537174	Benzenoids	Benzene and substituted derivatives	up
76	Mix vs NS	[M-H]-	Rauwolscine	2.543841177	1.920167601	0.005435784	/	/	up
77	Mix vs NS	[M-H]-	Butyric acid	1.226731691	1.987632916	0.005845815	/	/	up
78	Mix vs NS	[M-H]-	Lumichrome	1.16602511	1.915411925	0.006105396	Organoheterocyclic compounds	Pteridines and derivatives	up
79	Mix vs NS	[M-H]-	Hydroquinidine	7.704739725	1.762703649	0.00616079	/	/	up
80	Mix vs NS	[M-H]-	Isoimperatorin	1.124436277	1.867497463	0.007315404	Phenylpropanoids and polyketides	Coumarins and derivatives	up
81	Mix vs NS	[M-H-H2O]-	16-phenyltetranor prostaglandin f2.alpha.	2.822449707	2.044087362	0.010706427	Lipids and lipid-like molecules	Fatty Acyls	up
82	Mix vs NS	[M-H]-	P-toluenesulfonic acid	1.958949697	1.885703804	0.011915821	Benzenoids	Benzene and substituted derivatives	up
83	Mix vs NS	[M-H]-	2,6’-dimethoxy-2’-hydroxychalcone	1.083032224	1.837169643	0.012480837	Phenylpropanoids and polyketides	Linear 1,3-diarylpropanoids	up
84	Mix vs NS	[M-H]-	Picein	1.066494669	1.885478178	0.012738134	Organic oxygen compounds	Organooxygen compounds	up
85	Mix vs NS	[M-H]-	9-phenyl-1-(2,4,6-trihydroxyphenyl)nonan-1-one	2.333494885	2.012294905	0.016825485	Organic oxygen compounds	Organooxygen compounds	up
86	Mix vs NS	[M-H]-	Pg 42:11	1.018145624	0.502101398	0.021620203	Lipids and lipid-like molecules	Glycerophospholipids	down
87	Mix vs NS	[M-H]-	Isovaleric acid	1.688616357	2.279264606	0.034684281	Lipids and lipid-like molecules	Fatty Acyls	up
88	Mix vs NS	[M-H]-	Cis-7,10,13,16-docosatetraenoic acid	2.009489827	1.419544777	0.035596707	Lipids and lipid-like molecules	Fatty Acyls	up
89	Mix vs NS	[M-H]-	3-hydroxybenzaldehyde	2.815179123	0.582474104	0.043606723	Organic oxygen compounds	Organooxygen compounds	down

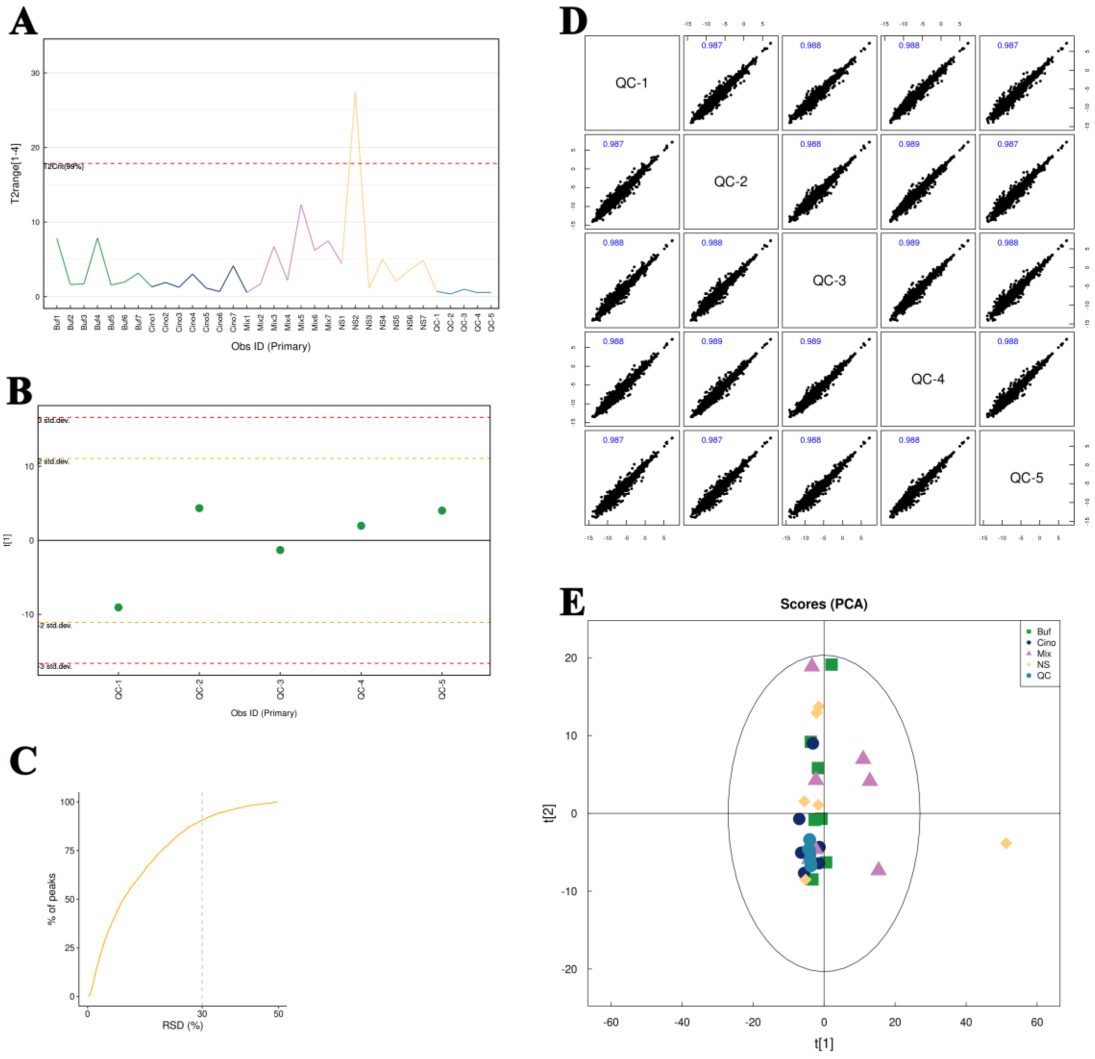

Figure S1. Results of quality control. **A** Hotelling's T2 test for the total sample in negative mode. **B** Multivariate control chart of QC samples in negative mode. **C** Relative standard deviation of QC samples in negative mode. **D** Pearson correlation analysis of QC samples in negative mode. **E** Principal component analysis of QC samples in negative mode.

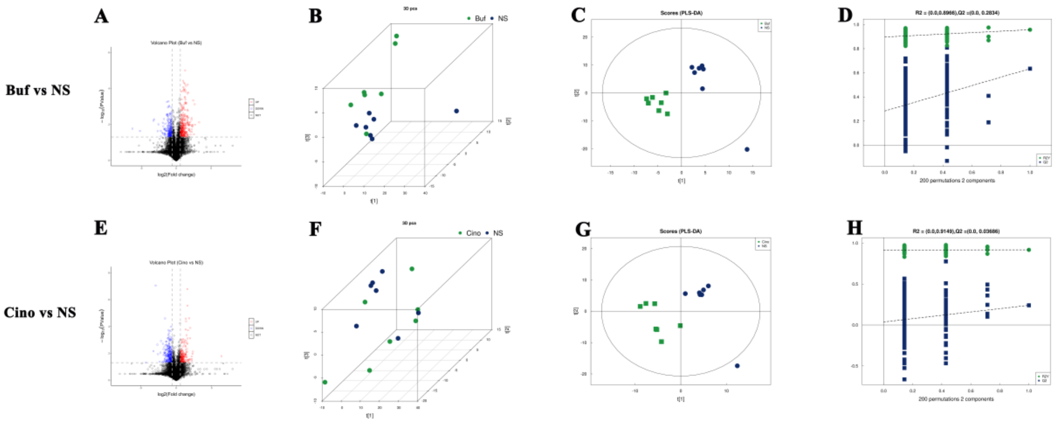

Figure S2. A and E Volcanograms showing up-and down-regulated metabolites between NS and Buf, NS and Cino group, respectively. Blue color indicates downregulated metabolites, while red color represents upregulated metabolites. **B and F** Score plot of 3D principal component analysis between NS and Buf, NS and Cino group, respectively. **C and G** Score plot of partial least-squares discriminant analysis between NS and Buf, NS and Cino group, respectively. **D and H** Corresponding validation plot between NS and Buf, NS and Cino group, respectively.

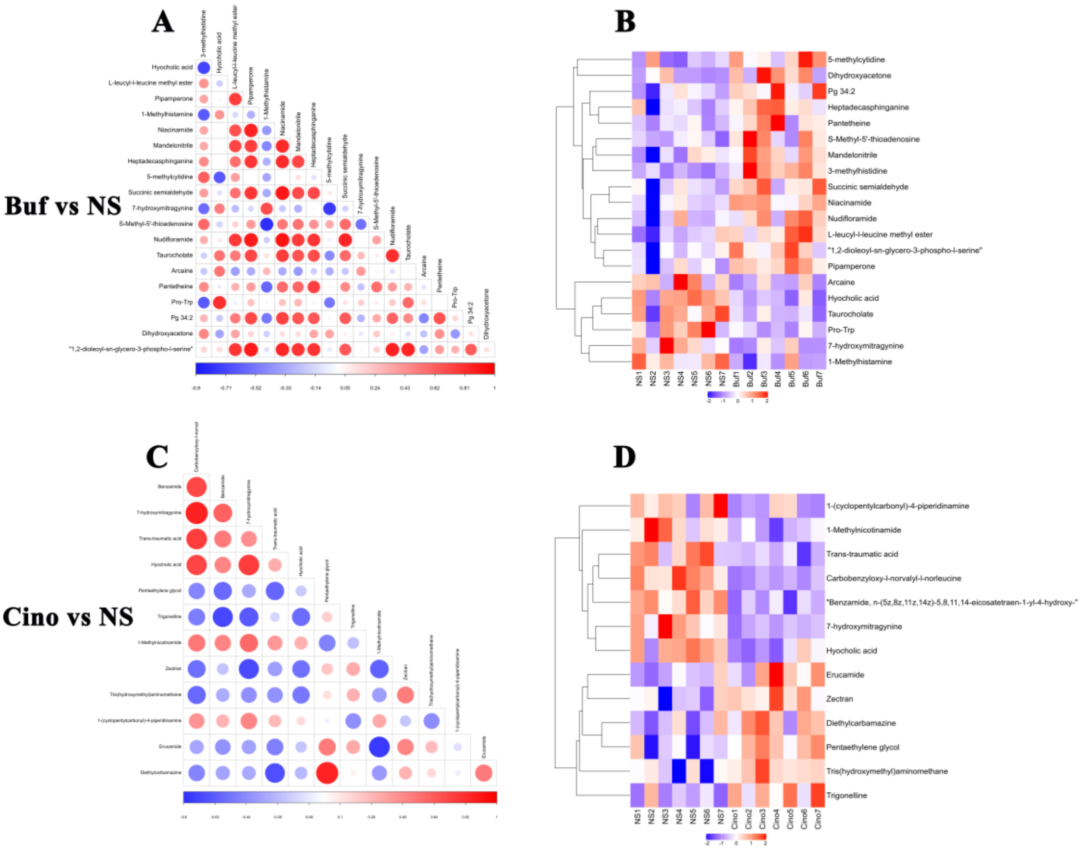

Figure S3. A and C Correlation analysis of differential metabolites between NS and Buf, NS and Cino group, respectively. Red color indicates positive correlation and blue color indicates negative correlation. The higher the value, the stronger the correlation between the two metabolites. **B and D** Heatmap analysis of differential metabolites between NS and Buf, NS and Cino group, respectively. Red color indicates higher concentration of differential metabolites, blue color indicates concentration of differential metabolites.

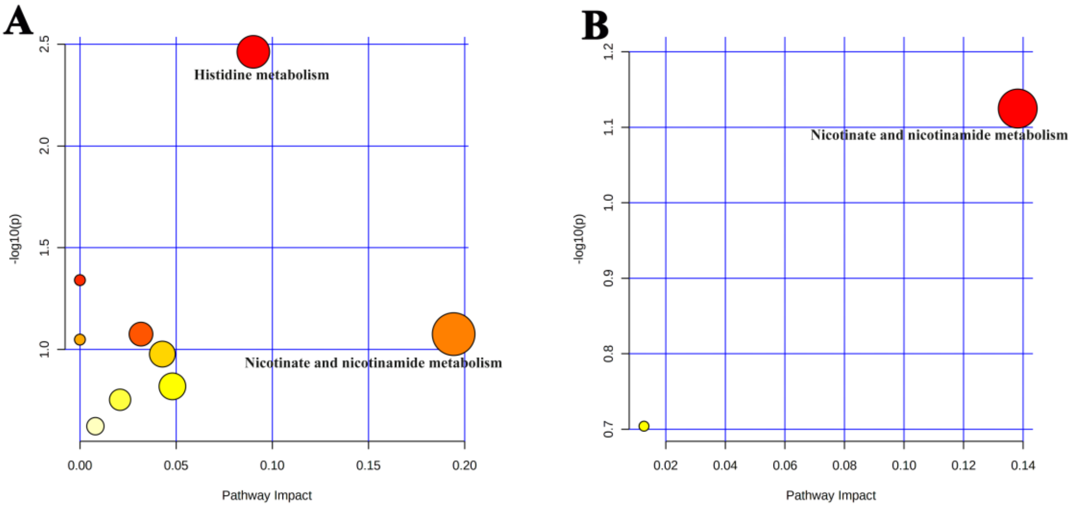

Figure S4. A-B Metabolic pathway of differential metabolites between NS and Buf, NS and Cino group enriched by MetaboAnalyst 5.0.
